# Supplementary material for: Investigating the influence of physiologically relevant hydrostatic pressure on CHO cell batch culture
Source: Sci Rep. 2021 Jan 8;11:162. doi: 10.1038/s41598-020-80576-8 (PMC7794228; doi:10.1038/s41598-020-80576-8)
Supplement: Supplementary file 1 — Supplementary Information 1. [file 41598_2020_80576_MOESM1_ESM.zip › S1/IgG/Feb17 60 90 standard/old/Feb13 90mmHg 3.pdf]

Data File C:\Users\P...mStation\1\Data\TK_CHO_IgG 2019-02-17 14-04-01\028-3-90mmHg Feb13.D
Sample Name: 90mmHg Feb13

===

Acq. Operator : SYSTEM   Seq. Line : 28
Sample Operator : SYSTEM
Acq. Instrument : HPLC-0039   Location : 3
Injection Date : 2/17/2019 4:00:42 PM   Inj : 1
                                    Inj Volume : 100.000 µl
Method : C:\Users\Public\Documents\ChemStation\1\Data\TK_CHO_IgG 2019-02-17 14-04-01 \CHO_IgG_LC.M (Sequence Method)
Last changed : 2/8/2019 3:17:23 PM by SYSTEM

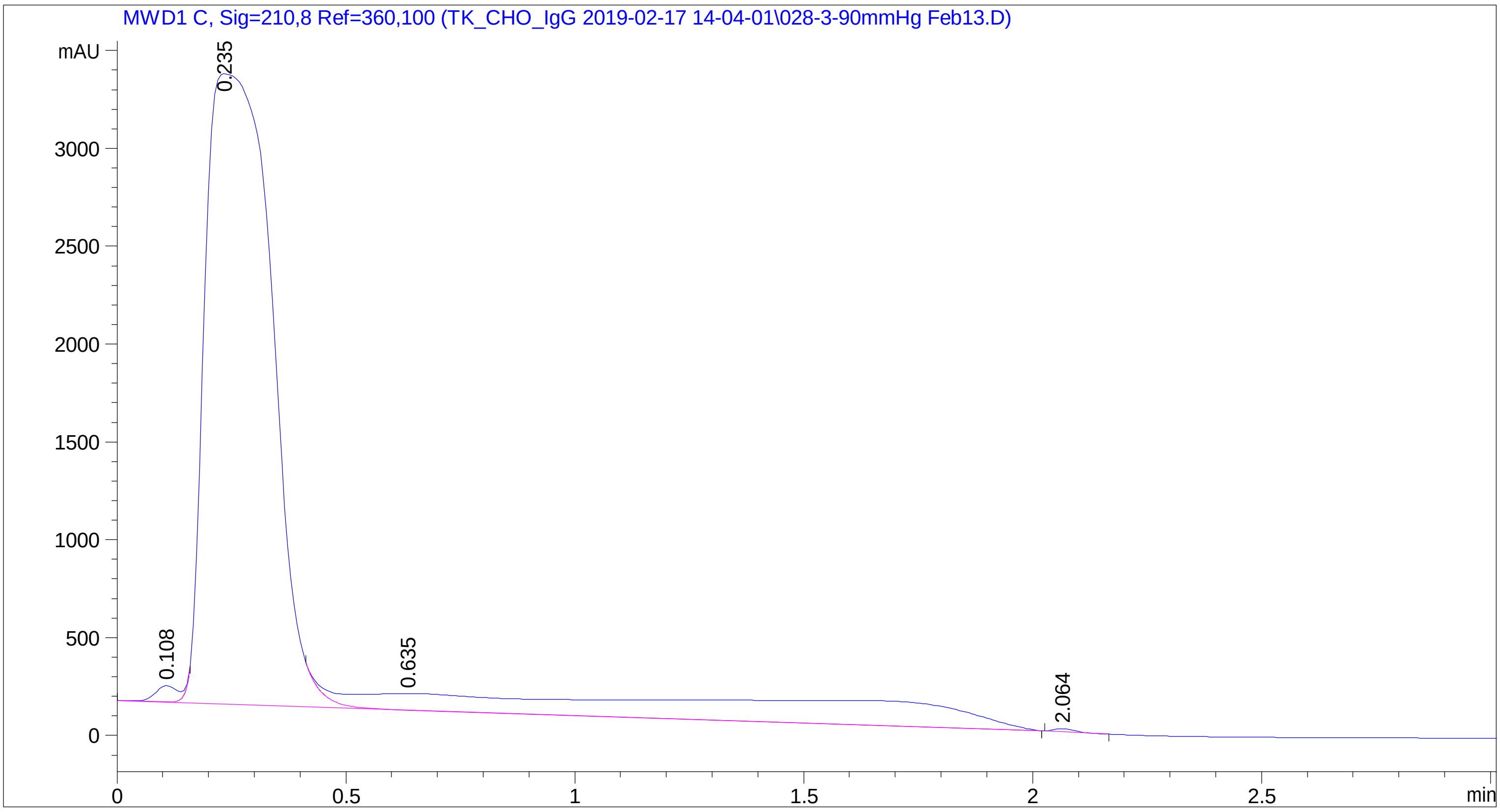

===
Area Percent Report
===

Sorted By : Signal
Multiplier : 1.0000
Dilution : 1.0000
Use Multiplier & Dilution Factor with ISTDs

Signal 1: MWD1 C, Sig=210,8 Ref=360,100

Peak #	RetTime [min]	Type	Width [min]	Area [mAU*s]	Height [mAU]	Area %
1	0.108	BV E	0.0520	288.76089	84.23462	0.7003
2	0.235	VV R	0.1679	3.25578e4	3221.32935	78.9601
3	0.635	VB E	1.1952	8335.72656	84.92029	20.2161
4	2.064	BBA	0.0477	50.91801	16.62601	0.1235

Totals : 4.12332e4 3407.11026

===

*** End of Report ***
